# IL-6 Impairs Myogenic Differentiation by Downmodulation of p90RSK/eEF2 and mTOR/p70S6K Axes, without Affecting AKT Activity

**DOI:** 10.1155/2014/206026

**Published:** 2014-05-21

**Authors:** Michele Pelosi, Manuela De Rossi, Laura Barberi, Antonio Musarò

**Affiliations:** ^1^Institute Pasteur Cenci-Bolognetti, DAHFMO-Unit of Histology and Medical Embryology, IIM, Sapienza University of Rome, Via A. Scarpa 16, 00161 Rome, Italy; ^2^Center for Life Nano Science@Sapienza, Istituto Italiano di Tecnologia, Viale Regina Elena 291, 00161 Rome, Italy

## Abstract

IL-6 is a multifaceted pleiotropic cytokine, which is produced by a variety of cell types and targets different cells and tissues. In physiological conditions, IL-6 can be locally and transiently produced by skeletal muscle and plays an important role in muscle homeostasis. Circulating IL-6 levels are normally very low or undetectable but are dramatically increased in several pathologic conditions. In this study, we aimed to define the potential molecular mechanisms underlying the effects of IL-6 on myogenic program. We explored the molecular mechanisms through which exogenous IL-6, or the conditioned medium from the murine C-26 adenocarcinoma cells (a cellular model that secretes high levels of IL-6 and induces cancer cachexia in mice), interferes with the myogenic program. Our study revealed that IL-6 induces the activation of the Stat3 signaling and promotes the downmodulation of the p90RSK/eEF2 and mTOR/p70S6K axes, while it does not affect the activation of AKT. We thus identified potential molecular mediators of the inhibitory effects of IL-6 on myogenic program.

## 1. Introduction

Muscle differentiation is a well-coordinated and regulated process which is influenced by either positive or negative external signals. Fibroblast growth factor (FGF) and TGF-*β* inhibit differentiation [1, 2], whereas IGFs are potent inducers of myogenic proliferation, differentiation, and hypertrophy [3, 4].

Moreover, several cytokines and other factors, such as IL-1, IL-6, TNF-*α*, and IFN-*γ*, have been proven to negatively affect muscle differentiation both in vitro and in vivo, causing also muscle wasting [5–8]. Elevated levels of cytokines, associated with chronic inflammation, are observed in several chronic diseases, ranging from cancer to AIDS and from muscular dystrophy to chronic heart failure and kidney disease.

Among these factors, IL-6 potentially plays a prominent role in muscle differentiation, homeostasis, and wasting.

IL-6 can be produced locally, by skeletal muscle, or released systemically in response to different physiopathologic stimuli [9]. In skeletal muscle IL-6 acts as a pleiotropic factor and appears to have at least two different conflicting functions. In nonpathological conditions, IL-6 has been involved in the physiological metabolic response of skeletal muscle to exercise [9, 10]; it is synthesized by skeletal muscle, in response to increased workload, and secreted locally or into the blood stream, where it might act in a hormone-like manner, stimulating the hepatic gluconeogenesis and increasing adipose tissue lipolysis [10]. It has been also shown that the autocrine production of IL-6 can promote myogenic differentiation [11, 12] and acts as a positive regulator of satellite cell-mediated hypertrophic muscle growth [13], suggesting that IL-6 plays an important role in myogenesis.

By contrast, different pathologic conditions promoting muscle wasting, including cancer, are associated with elevated systemic levels of IL-6 [4]. Moreover, severe muscle atrophy is seen in different mouse models in which IL-6 has been chronically elevated in skeletal muscle, through transgenic overexpression [14], in vivo electroporation [15], or direct infusion into muscle [16]. The different and opposite effects of a single molecule, like IL-6, can be explained taking into consideration the levels of expression and its spatial and temporal interaction with the target tissue. The regulation of muscle differentiation is dependent on the activation of signal transduction cascades with the complex involvement of several kinases, including the mitogen-activated protein kinases (MAPK), the serine/threonine kinase AKT, a downstream effector of PI3-kinase, the p90 ribosomal S6 kinase (p90RSK), and the calcineurin/NFAT pathway [17].

In this study, we explored some molecular mechanisms through which exogenous IL-6 can perturb the skeletal muscle homeostasis and impinge muscle differentiation.

We treated C2C12 myogenic cells, an in vitro model able to recapitulate the program of myogenic differentiation with recombinant IL-6 or with the conditioned medium from the C-26 mouse adenocarcinoma cell line (C-26 CM), which secrete IL-6 in the extracellular medium (as it is shown in this paper and in [5, 18]). We show that IL-6 induced a dramatic inhibition in the expression of late myogenic differentiation markers, such as myogenin and sarcomeric muscle myosin, whereas it did not significantly affect the expression of Pax7 and MyoD. IL-6 also did not affect the activation of AKT, a kinase essential to promote protein synthesis and cell survival and to block protein degradation [19, 20]. However, we show that exogenous IL-6 induced a selective downmodulation of the p90RSK/eEF2 and mTOR/p70S6K axes, which are important mediators for the positive control of protein synthesis and the maintenance of the differentiated myogenic phenotype [19, 21–23].

## 2. Material and Methods

### 2.1. Cell Culture and Media Preparation

Murine C2C12 myoblasts cells and C-26 colon adenocarcinoma (ATCC) were grown in Dulbecco's modified Eagle's medium (DMEM) with 10% fetal bovine serum plus standard complements (growth medium, GM). To induce myogenic differentiation of C2C12 myoblasts, cells were cultured in GM until subconfluence (i.e., about 80–90% of cell confluence) and then shifted to DMEM with 2% horse serum (differentiation medium, DM). All media, sera, and complements for cell cultures were from Gibco-Invitrogen. For C-26 conditioned medium (CM) production, about 8 × 10^5^ C-26 cells were plated in GM in a 60 × 15 mm cell culture dish (Falcon). After the overnight attachment, the medium was totally replaced with 6 mL of fresh GM. When the C-26 cells reached confluence, the resulting medium (C-26 CM) was collected, centrifuged (1000 g for 10 min), and filtered with a 0.22 *μ*m polyethersulfone hydrophilic filter (Millipore). 2% horse serum (final concentration) was added to C-26 CM, which was finally diluted in a ratio 1 : 10 in the DM, to obtain the DM + 10% C-26 CM medium.

Recombinant mouse IL-6 (rIL-6) was from R&D Systems. Lyophilized rIL-6 was dissolved at 100 *μ*g/mL in sterile PBS containing 0.1% bovine serum albumin (BSA, globulin-free and low endotoxin, purity ≥98%, from Sigma). rIL-6 was diluted into DM to obtain the (DM + 25 ng/mL of rIL-6) medium. The same amount of carrier BSA was added in the control DM. However, previous analysis showed that the carrier BSA did not interfere with myogenic differentiation (data not shown).

### 2.2. Cytokine Assay

The BD Biosciences CBA Mouse Inflammation Kit was used to quantitatively measure IL-6, IL-10, MCP-1, IFN-*γ*, TNF-*α*, and IL-12p70 protein levels, according to the manufacturer instructions. In brief, 50 *μ*L of supernatants (C-26 CM or control samples) were incubated with a mixture of beads coated with the capture antibodies for the different cytokines. Addition of the PE-conjugated detection antibodies formed a sandwich complex. After 2 h of incubation and one wash, samples were analyzed by flow cytometry, using an Epics XL Cytometer (Beckman Coulter). Standard curves were generated from analysis of titrated cytokine standards (at least 10 serial dilutions; standards provided by the manufacturer) and analysis was performed using the BD CBA analysis software.

### 2.3. Immunohistochemical Analysis

C2C12 myotubes were fixed in 4% paraformaldehyde and then preincubated in phosphate-buffered saline (PBS) containing 1% BSA and a 1 : 30 dilution of goat serum for 30 min. Myotubes were treated with a monoclonal antibody against the anti-myosin type II (MF-20, Developmental Studies Hybridoma Bank) (1 : 100, overnight at 4°C). After incubation with the primary antibody, the myotubes were washed twice with PBS and once with PBS containing 1% BSA for 15 min and incubated with goat anti-mouse rhodamine-conjugated secondary antibody. The cells were washed again, as described above, and the nuclei of myogenic cells were visualized by Hoechst staining. Finally the slides were mounted in 90% glycerol in PBS (pH 8) and visualized under inverted microscope (Axioskop 2 plus; Carl Zeiss MicroImaging, Inc.) and images were processed using Axiovision 3.1.

### 2.4. Protein Extraction and Immunoblotting

C2C12 cells were washed with PBS and lysed in RIPA buffer (radio immunoprecipitation assay buffer) (100 mM Tris/HCl pH 7.4, 150 mM NaCl, 20 mM sodium fluoride, 2 mM EDTA, 0.2 mM 2-glycerophosphate, 1 mM sodium vanadate, 3% (w/v) deoxycholate, 10 mM Na_4_P_2_O_7_, 0.5% SDS, and 1% (v/v) Nonidet P40) containing a protease inhibitor cocktail (Complete Mini, Roche). Cells were sonicated and cleared by centrifugation (10,000 g for 10 min, at 4°C), and the whole cell lysate supernatant was collected. Proteins were resolved by SDS/PAGE, transferred on to Protran nitrocellulose membranes (Whatman), and immunoblotted under standard conditions, with primary antibodies as follows: anti-myosin type II MF-20 (Developmental Studies Hybridoma Bank); anti-*α*-tubulin (Sigma); anti-phospho-Stat3 (Tyr705) D3A7 (Cell Signaling Technology); anti-Stat3 D3Z2G (Cell Signaling); anti-phospho-AKT (Ser473) D9E (Cell Signaling); phospho-Akt (Thr308) (D25E6); anti-AKT (Cell Signaling); anti-phospho-mTOR (Ser2448) D9C2 (Cell Signaling); anti-mTOR (Cell Signaling); anti-phospho-p70S6 kinase (Thr389) 108D2 (Cell Signaling); anto-phospho-p70 S6 Kinase (Ser371) (Cell Signaling); anti-p70S6 Kinase (Cell Signaling); anti-phospho-p90RSK (Thr359/Ser363) (Cell Signaling); anti-pan p90RSK number 9347 (Cell Signaling); anti-eEF2 (Thr56) (Cell Signaling); and anti-eEF2 (Cell Signaling). Anti-mouse or anti-rabbit immunoglobulin G horseradish peroxidase linked F(ab′)2 secondary antibodies and enhanced chemiluminescence detection kit were from Amersham Biosciences.

### 2.5. RNA Isolation, RT (Reverse Transcription), and Real Time PCR

C2C12 cells were washed with ice-cold PBS and total RNA was extracted using the TRIzol reagent (Life Technologies). Digestion of contaminating genomic DNA was performed with DNA-free DNase treatment (Ambion). First strand cDNA was generated using a kit with random primers (High Capacity cDNA Reverse Transcription kit, Applied Biosystems) from 1 *μ*g of total RNA. Newly synthesized cDNA was diluted 5-fold in DNA-free water and 5% of this cDNA was used in each real-time PCR assay, using a 7500 Real Time PCR System (Applied Biosystems). The standard curve method was used to calculate the relative mRNA levels for each transcript examined and actin beta (ACTB) was used as a reference to normalize the data. Specific assays used for the Taqman quantification were all from Applied Biosystems: MYOG: Mm00446195_g1; MYOD1: Mm01203489_g1; PAX-7: Mm01354484_m1; SOCS3: Mm00545913_s1; ACTB: Mm00607939_s1.

### 2.6. Densitometric Analysis and Statistics

Densitometry was performed on scanned immunoblot images (Aida 2.11 software). The gel analysis tool was used to obtain the absolute intensity (AI) for each experimental band and corresponding control band. Relative intensity (RI) for each experimental band was calculated by normalizing the experimental AI to the corresponding control AI. In both densitometric analysis and real-time PCR analysis, the software GraphPad Prism was used to perform statistical analysis with Student's *t*-test. Results are expressed as means ± s.e.; *P* values <0.05 were considered statistically significant.

## 3. Results

### 3.1. C-26 Adenocarcinoma Cells Secrete IL-6

**Figure 1:**
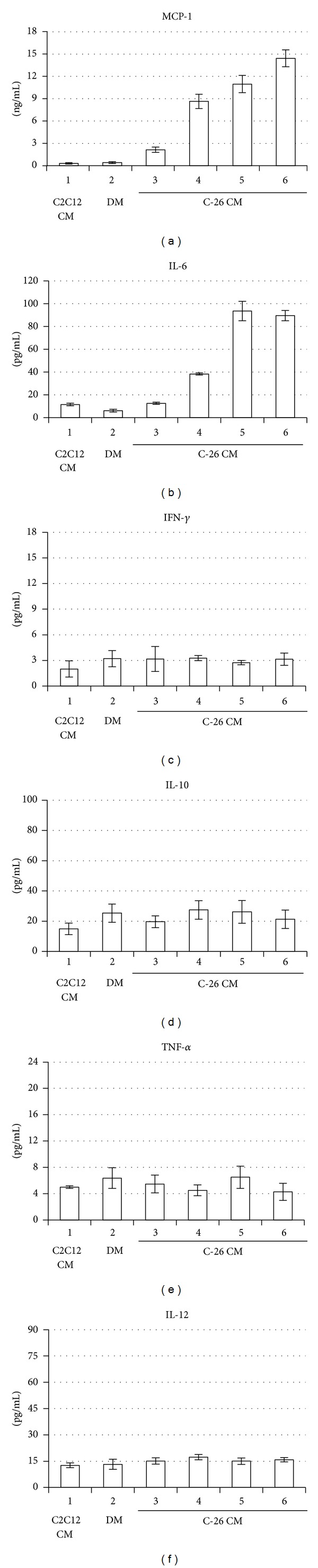
Determination of secreted cytokines in the conditioned medium from C-26 adenocarcinoma cells. (a–f) MCP-1, IL-6, IFN-*γ*, IL-10, TNF-*α*, and IL-12p70 protein levels were measured using a cytometric bead assay in which the specific antibodies were conjugated with capture beads (see Section 2). MCP-1 concentration is expressed in ng/mL, whereas the other cytokines concentrations are in pg/mL. 1: control I, conditioned medium (CM) from proliferating C2C12 cells in growth medium (GM); 2: control II, myogenic differentiation medium (DM); 3: CM from proliferating C-26 cells in GM (i.e., exponential phase, with a cell confluence about 50%); 4, 5, and 6: CM from confluent C-26 cells (i.e., stationary phase) with a cell confluence of about 100% for 1, 2, or 3 days, respectively. The experiment indicates means ± standard deviation from three independent determinations.

Proinflammatory cytokines might impinge muscle differentiation both in vitro and in vivo [6–8]. We measured the concentration of IL-6, IL-10, TNF-*α*, MCP-1, INF-*γ*, and IL-12p70 secreted in the conditioned medium (CM) by the murine colon C-26 adenocarcinoma cells [5, 18]. This quantitative analysis revealed that C-26 cells secreted and progressively accumulated, in CM, relatively high concentrations of two cytokines, IL-6 and MCP-1 (Figure 1). The levels of the other cytokines secreted in CM by C-26 cells, such as INF-*γ*, IL-10, TNF-*α*, and IL-12, were not significantly different compared with the control medium obtained from the C2C12 myogenic cell line (Figure 1).

### 3.2. C-26 Conditioned Medium Inhibits C2C12 Myogenic Differentiation

**Figure 2:**
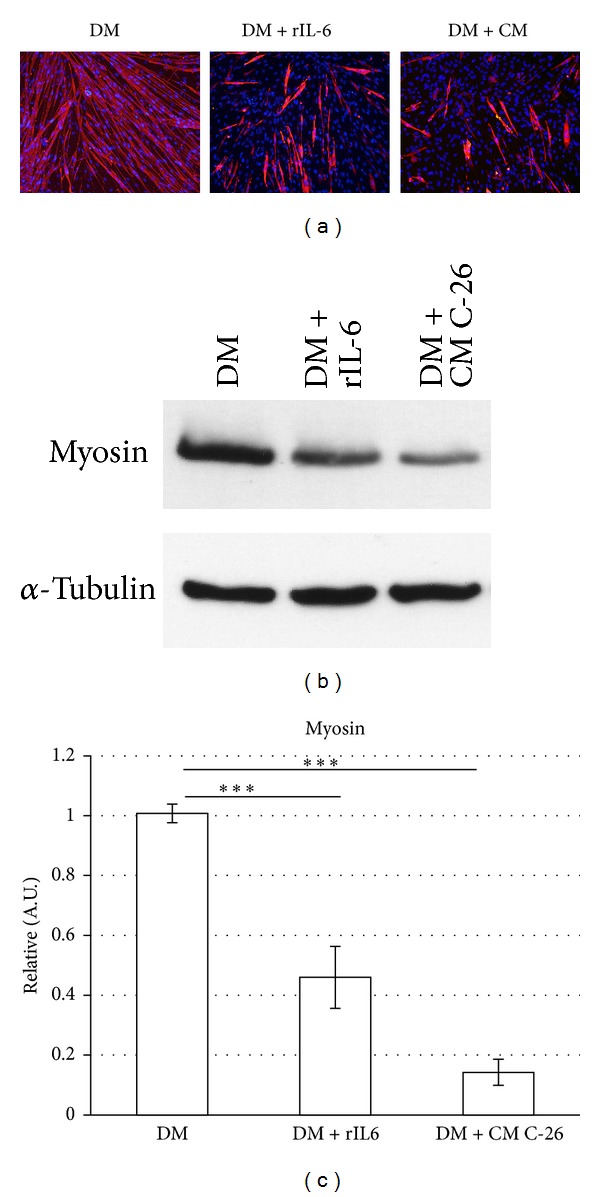
C-26 CM and exogenous IL-6 impair the myogenic differentiation of C2C12 cells. C2C12 myoblasts were cultured in GM until subconfluence and then shifted to differentiation medium (DM), or DM containing 10% C-26 CM, or DM containing 25 ng/mL of recombinant IL-6. (a) After 48 h in the differentiation media, cells were stained using an anti-sarcomeric myosin heavy chain (MF-20) antibody (red) and the Hoechst staining of the DNA (blue) and was imaged by conventional fluorescent microscopy. (b) After 48 h in the differentiation media, cells were harvested and total protein was extracted; cell lysates (70 *μ*g of total protein) were subjected to Western blot analysis with the indicated specific antibodies. The experiment shown is representative of three independent determinations. (c) Densitometric analysis of the relative protein levels (obtained from Western blotting analysis) of sarcomeric myosin heavy chain, represented as arbitrary units (A.U.) and equalized for *α*-tubulin expression. ***P < 0.005 (Student's *t*-test).

In agreement with previously published experiments [24], we observed that when C-26 CM was added to the standard growth medium, C2C12 myoblasts proliferation was impaired (data not shown). To explore if the factors secreted by C-26 adenocarcinoma cells were able to directly interfere with muscle differentiation, we let proliferate C2C12 myoblasts in the standard growth medium until about 80–90% of cell confluence (subconfluence) and then we shifted the cells to the differentiation medium (DM) containing 10% C-26 CM (Figure 2(a)).

Morphological and immunofluorescence analysis revealed that the presence of C-26 CM in DM induced a significant reduction in the number and size of the myosin positive multinucleated myotubes, compared with control C2C12 myotubes (Figure 2(a)). The altered differentiated muscle phenotype was also confirmed by Western blot analysis, revealing a drastic downmodulation of the sarcomeric myosin heavy chain expression, a specific marker of myogenic differentiation (Figures 2(b) and 2(c)).

Our results demonstrate that factors released in the extracellular medium by C-26 adenocarcinoma cells were able to interfere, in a cell autonomous manner, with C2C12 myogenic differentiation and maturation.

### 3.3. IL-6 Negatively Regulates C2C12 Myogenic Differentiation

Previous studies have shown that C-26 conditioned medium is a complex mixture of secreted proteins, including cytokines, growth factors, and signaling molecules, some of which can potentially play a role in myogenic program and muscle wasting [5, 24]. In this work, using a proinflammatory cytokine array, we have shown that adenocarcinoma C-26 cells can secrete and progressively accumulate in the CM high concentrations of at least two cytokines, IL-6 and MCP-1 (Figure 1).

We explored if IL-6 can recapitulate the inhibitory effect of C-26 CM on C2C12 myogenic differentiation or whether this inhibition is the sum of the concomitant effects of several tumoral factors secreted by C-26 cells.

To avoid any confounding effect, we induced C2C12 myogenic differentiation in the presence of the differentiation medium (DM) supplemented with recombinant exogenous IL-6 protein (rIL-6). We then analysed the differentiated myotubes by Western blotting and immunofluorescence analysis (Figure 2). We observed that the C2C12 myotubes treated with rIL-6 were shorter and smaller in size (Figure 2(a)) and displayed a significant reduction in the sarcomeric myosin heavy chain expression compared with the myotubes differentiated in the control medium (DM) (Figures 2(b) and 2(c)).

Of note, the inhibitory effects of rIL-6 and C-26 CM on C2C12 myogenic differentiation were similar (Figures 2(a)–2(c)).

**Figure 3:**
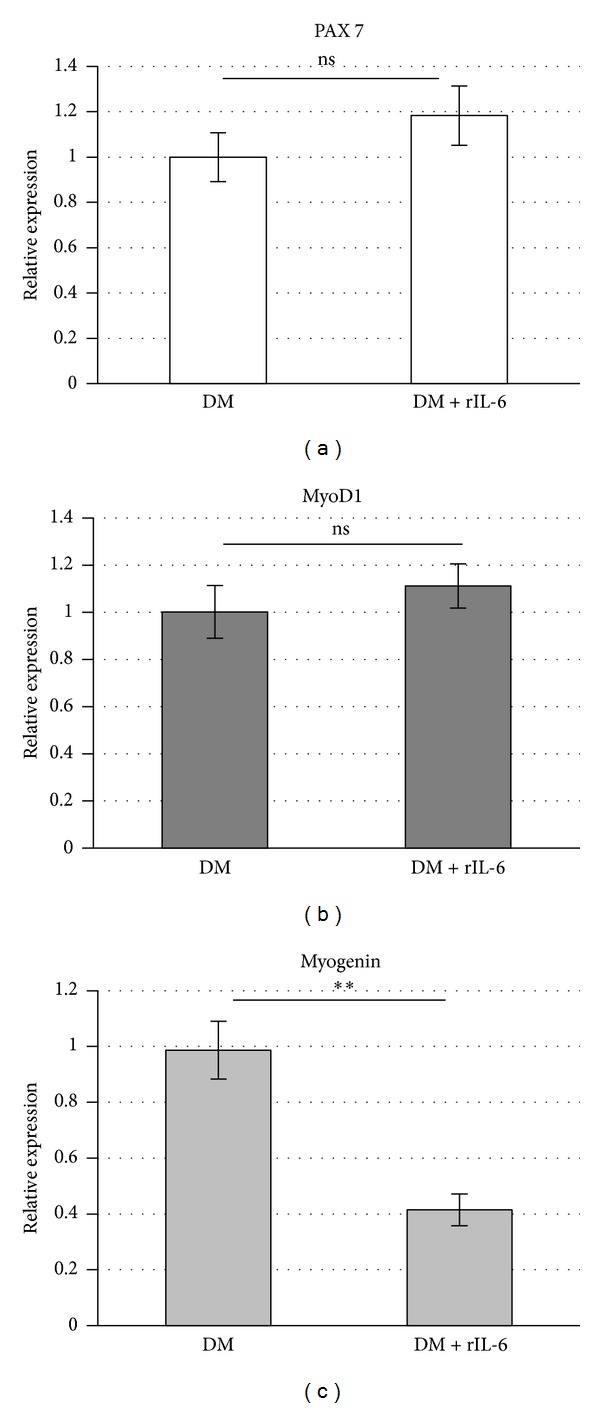
Exogenous IL-6 impairs the expression of myogenin, without affecting the expression of Pax-7 and MyoD. C2C12 myoblasts were cultured in GM until subconfluence and then shifted to DM or DM containing 25 ng/mL of recombinant IL-6. After 48 h in the differentiation media, cells were harvested, total RNA was extracted, and first-strand cDNA was generated from 1 *μ*g of total RNA to perform real-time PCR using specific Taqman probes. Expression data were normalized using actin beta (ACTB) as housekeeping gene. Results are expressed as means ± standard deviations. **P < 0.005; n.s., not significant (Student's *t*-test).

We also analyzed, by real-time PCR, the effects of rIL-6 on the expression of Pax-7 and MyoD1, two transcription factors that are expressed prevalently in the proliferating myoblasts and in the early stages of the myocytes differentiation [25], and of myogenin, a molecular marker associated with the myocytes' full commitment to differentiation [25] (Figure 3). rIL-6 did not induce any significant change in Pax7 and MyoD expression (Figures 3(a) and 3(b)), whereas we observed a significant downregulation of the myogenin expression compared with controls (Figure 3(c)). On the whole, these data show that exogenous rIL-6 negatively regulates C2C12 myogenic differentiation, without interfering with the early stage of the myogenic program.

### 3.4. Exogenous IL-6 Activates Stat3-SOCS3 Signaling during C2C12 Myogenic Differentiation

Skeletal muscle expresses the IL-6 receptor (IL6R/CD126) and it is a target of the IL-6 activity [10]. Binding of IL-6 to its receptor initiates specific intracellular events, including phosphorylation and activation of the Janus kinases (JAK) and the subsequent activation of the signal transducer and activator of transcription (Stat) [26, 27]. IL-6-induced Stat signaling also initiates a negative feedback regulation through the increased transcription of the suppressor of cytokine signaling (SOCS) proteins [27].

**Figure 4:**
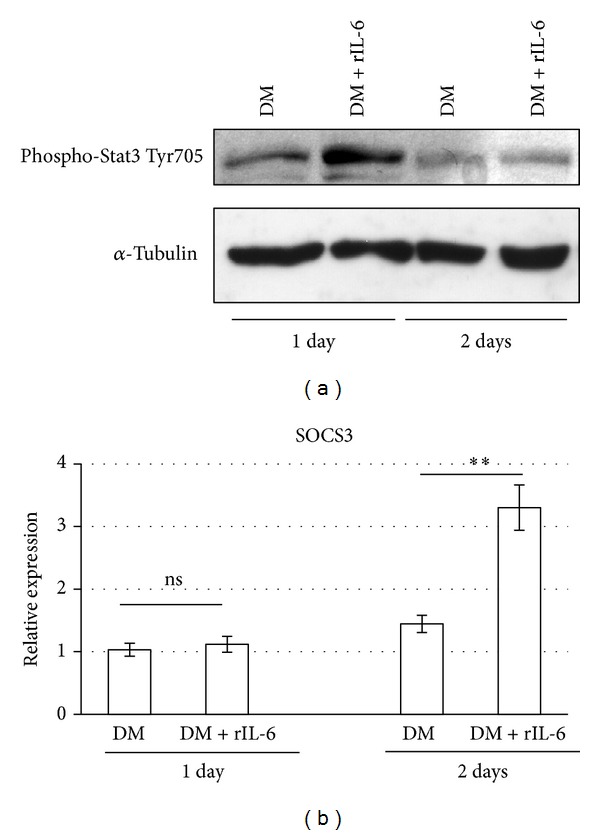
Exogenous IL-6 activates Stat3-SOCS3 signaling during myogenic differentiation. C2C12 myoblasts were cultured in GM until subconfluence and then shifted to DM or DM containing 25 ng/mL of recombinant IL-6. After 24 h or 48 h in the differentiation media, cells were harvested and total protein was extracted, or total RNA was extracted and first-strand cDNA was generated from 1 *μ*g of total RNA. (a) Cell lysates (70 *μ*g of total proteins) were subjected to Western blotting analysis with the indicated specific antibodies. The experiment shown is representative of three independent determinations. (b) Real-time PCR was performed on cDNA, using the indicated specific Taqman probes. Expression data were normalized using actin beta (ACTB) as housekeeping gene. The experiment shows means ± standard deviation of three independent determinations. **P < 0.005; n.s., not significant (Student's *t* test).

We analyzed whether rIL-6 treatment activates the canonical signaling through the IL-6 receptor. To this purpose, we induced C2C12 myogenic differentiation in the presence of the differentiation medium (DM) supplemented with rIL-6. Western blot analysis showed that rIL-6 treatment elicited a significant upregulation of the active phosphorylated form (on the Tyr 705 residue) of Stat3 (phospho-Stat3) at the early stages of myogenic differentiation (i.e., day 1; Figure 4(a)), whereas rIL-6 did not induce significant changes in phospho-Stat3 at later stages of the myogenic differentiation (i.e., day 2 and later; Figure 4(a) and data not shown). Real-time PCR analysis showed that the expression of SOCS3 (the suppressor of the IL-6 signaling) was induced by rIL-6 treatments only at the late stage of the myogenic differentiation (i.e., day 2 and later; Figure 4(b) and data not shown).

In conclusion, this experiment shows that in C2C12 cells the exogenous rIL-6 is able to efficiently activate the Stat3-SOCS3 signaling during myogenic differentiation; moreover, the cytokine-inducible positive activation of Stat3 takes place at the early stages of myogenic differentiation, whereas the negative feedback of the signaling pathway (promoted by the Stat signaling itself) is activated at later differentiation stages, through the transcriptional induction of SOCS3.

### 3.5. Exogenous IL-6 Perturbs mTOR and p70S6K Activities, without Affecting AKT Activity, during C2C12 Myogenic Differentiation

AKT inhibition can substantially contribute to impinge muscle differentiation and muscle atrophy [19, 20, 28]. AKT plays a central role in the control of both muscle protein synthesis, via mTOR, and protein degradation, via the transcription factors of the FoxO family [19, 20].

**Figure 5:**
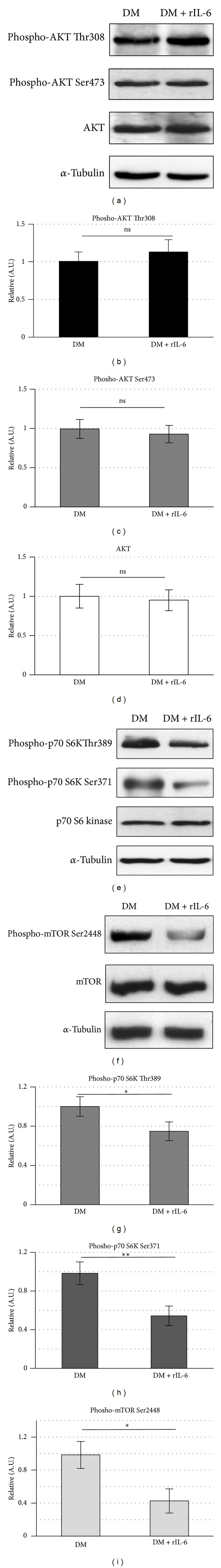
IL-6 perturbs mTOR and p70S6K activities, without affecting AKT activity. C2C12 myoblasts were cultured in GM until subconfluence and then shifted to DM or DM containing 25 ng/mL of recombinant IL-6. (a, e, and f) After 48 h in the DM, cells were harvested and total protein was extracted; cell lysates (70 *μ*g of total proteins) were subjected to Western blotting analysis with the indicated specific antibodies. Each experiment shown is representative of three independent determinations. (b, c, d, g, h, and i) Densitometric analysis of the relative protein levels (obtained from Western blotting analysis), represented as arbitrary units (A.U.) and equalized for total AKT expression (b and c), *α*-tubulin expression (d), total p70S6K expression (g and h), or total mTOR expression (i). The experiments show means ± standard deviation of three independent determinations. n.s., not significant; *P < 0.05; **P < 0.005 (Student's *t* test).

Thus, we analyzed if exogenous rIL-6 could perturb AKT signaling pathways during C2C12 myogenic differentiation. We induced C2C12 differentiation in the presence of DM supplemented with rIL-6 and then analyzed the differentiated myotubes by Western blotting using specific antibodies (Figure 5). In the C2C12 myotubes differentiated in the presence of rIL-6 we did not observe any significant change in the levels of both the total AKT protein and the phosphorylated/active forms of AKT, namely, phospho-AKT Thr308 and phospho-AKT Ser473 (Figures 5(a)–5(d)). However, rIL-6 induced a significant reduction in the phosphorylated/active forms of mTOR (phospho-Ser2448) and p70S6K (phospho-Thr389 and phospho-Ser371) (Figures 5(e)–5(i)). The 2 kinases mTOR and p70S6K are among the crucial downstream effectors of the AKT pathway and positively control protein synthesis and muscle differentiation [29].

In conclusion, this experiment shows that, during C2C12 myogenic differentiation, AKT does not sustain alone the activity of mTOR and p70S6K1. We suggest that an ancillary pathway should contribute to sustain the activity of mTOR and p70S6K during C2C12 myogenic differentiation and when this ancillary pathway is inhibited, by exogenous rIL-6, the activation of mTOR and p70S6K is perturbed and the myotubes formation is impaired (see Figures 2 and 5).

### 3.6. Exogenous IL-6 Affects the Phosphorylation Levels of p90RSK and eEF2 during C2C12 Myogenic Differentiation

**Figure 6:**
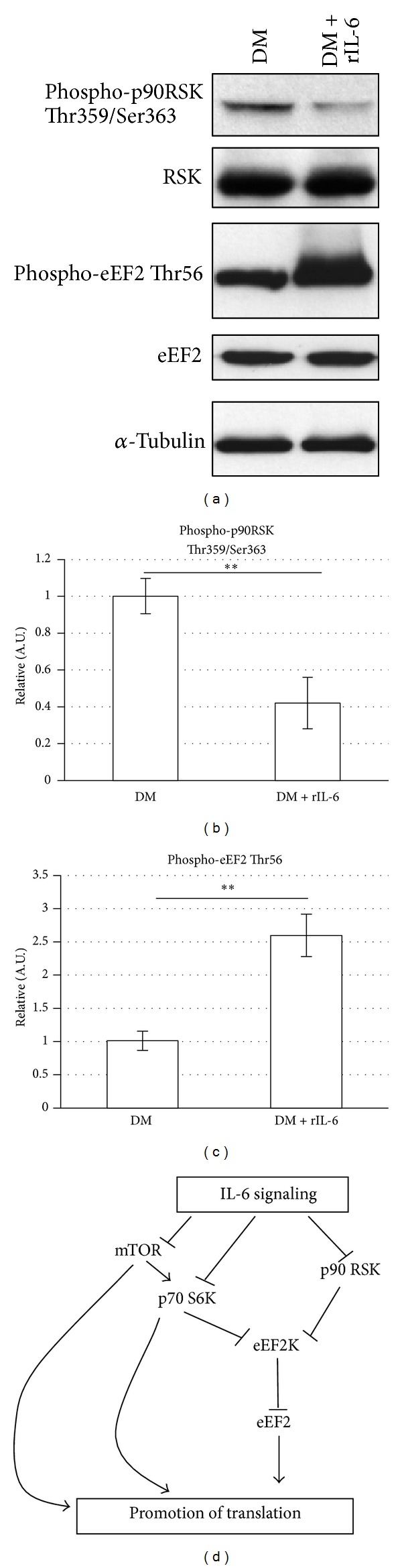
IL-6 affects the phosphorylation levels of p90RSK and eEF2. C2C12 myoblasts were cultured in GM until subconfluence and then shifted to DM or DM containing 25 ng/mL of recombinant IL-6. (a and c) After 48 h in the DM, C2C12 cells were harvested and total protein was extracted; cell lysates (70 *μ*g of total proteins) were subjected to Western blotting analysis with the indicated specific antibodies. Each experiment shown is representative of three independent determinations. (b and d) Densitometric analysis of the relative protein levels (obtained from Western blotting analysis), represented as arbitrary units (A.U.) and equalized for total RSK (b) or total eEF2 (c). The experiments show means ± standard deviation of three independent determinations. **P < 0.005 (Student's *t*-test). (e) A schematic model of the signaling connections was highlighted and discussed in this study.

It has been demonstrated that, similar to AKT, signaling generated from p90RSK plays a positive role in the regulation of protein synthesis during myogenic differentiation and maturation [21, 22]. Active p90RSK increases the levels of the dephosphorylated (active) form of eEF2, which catalyzes the translocation of peptidyl-tRNA from the A site to the P site on the ribosome [21]. Moreover, p90RSK can promote mTOR and p70S6K1 activation independently from AKT (Figure 6(d) and [23]). Thus, we explored if IL-6 could perturb p90RSK signaling pathways during C2C12 myogenic differentiation (Figure 6). We induced C2C12 differentiation in the presence of DM supplemented with rIL-6. IL-6 was able to induce a negative regulation of p90RSK signaling in the myogenic cells. As shown in Figures 6(a) and 6(b), p90RSK phosphorylation/activation was significantly downregulated in C2C12 differentiating cells treated with rIL6, and the reduced p90RSK active form was associated with a significant increase in the levels of the eEF2 phosphorylation (Figures 6(a)–6(c)), which is involved in the downmodulation of the translocation step in the protein synthesis [21].

In summary, in this study we suggest that, during C2C12 myogenic differentiation, exogenous rIL-6 can induce a depression of the protein synthesis in the myotubes, via a concomitant downmodulation of the p90RSK/eEF2 axis and of the mTOR/p70S6K, without apparently affecting the AKT activity.

## 4. Discussion

There is increasing evidence demonstrating the critical role of IL-6 in muscle homeostasis, regeneration, and differentiation [9]. Mouse models in which IL-6 has been locally chronically elevated in skeletal muscle, through transgenic overexpression [14], in vivo electroporation [15], or being infused directly into muscle [16], induced muscle atrophy. Finally, it has been shown that administration of IL-6 neutralizing antibodies or IL-6 inhibitors can reduce muscle wasting in tumor-bearing mice [5, 30].

In this study, we have shown that the murine colon C-26 adenocarcinoma cell line (C-26) secreted in the extracellular medium significant concentrations of two cytokines, IL-6 and MCP-1, whereas we could not detect any significant accumulation of other relevant inflammatory cytokines tested, namely, INF-*γ*, IL-10, TNF-*α*, and IL-12 (Figure 1).

The accumulation of the proinflammatory cytokine IL-6 in the extracellular medium of the C-26 cancer cells was not unexpected. Strassmann et al., in fact, reported that some clones of C-26 adenocarcinoma cells can secrete IL-6 and linked this cytokine to the development of a severe cachexia associated with muscle wasting [5]. It has been shown that mice implanted with the C-26 cells have an increased circulating IL-6 concentration, which coincides with muscle wasting [5].

In this study, we found that, in addition to IL-6, C-26 cells also secreted and accumulated in the extracellular medium the monocyte chemotactic protein-1 (MCP-1). MCP-1 is a chemokine and member of the small inducible cytokine family, playing a crucial role in the recruitment of monocytes and T lymphocytes into tissues [31]. In nonpathological conditions MCP-1 is expressed by adipocytes and by a number of other cell types, including smooth muscle and endothelial cells exposed to inflammatory stimuli [31]. MCP-1 has also been shown to play a prominent role as an inducer of insulin resistance in human skeletal muscle cells [32]. This latter role of MCP-1 may link directly this cytokine to the downmodulation of protein synthesis and/or to the increase in protein degradation and, in this way, to cancer cachexia. In this paper, we did not tackle the possible implication of MCP-1 on myogenic program, which will be addressed in a further dedicated investigation.

In our study, we aimed to explore the specific effect of IL-6 on the myogenic program, with the goal to demonstrate whether IL-6 was able to recapitulate the inhibitory effect of C-26 CM on C2C12 myogenic differentiation or whether this inhibition required the sum of the concomitant effects of several factors secreted by C-26 cells. Thus, to avoid any confounding result, we restricted the analysis to the proinflammatory cytokine IL-6. We added recombinant IL-6 (rIL-6) in the differentiation medium (DM) and we found that this cytokine is able to recapitulate the key inhibitory effects of C-26 CM on C2C12 myogenic differentiation. The presence of exogenous recombinant IL-6 in the DM induced a significant downregulation in the expression of the late markers of myogenic differentiation, like myogenin and the sarcomeric myosin heavy chain, whereas it did not induce any significant change in the expression of transcription factors that are expressed prevalently in the proliferating myoblasts and at the early stages of the myocytes differentiation [25] (Figure 2). This suggests that rIL-6 impinges muscle differentiation without hampering the early stage of the myogenic program.

We have also shown that the negative effects of rIL-6 on muscle differentiation are apparently mediated by the canonical activation of the Stat-SOCS signaling pathway (Figure 4). Our results are in accordance with a context in which IL-6 can play a prominent role in the cancer-induced muscle wasting [2, 4, 5, 11]. However, other reports have shown that in myogenic cells the autocrine production of IL-6 can also induce Stat3 activation but promotes the myogenic differentiation [11, 12]. It has been shown that IL-6 mRNA knockdown reduces muscle-specific gene expression in cultured C2C12 myoblasts [11], suggesting a potential myogenic role for the cytokine. Moreover, Serrano et al. identified IL-6 as an essential regulator of satellite cell-mediated hypertrophic muscle growth [13]. This apparent paradox can be justified considering the levels of expression of IL-6, its spatial and temporal interaction with the target tissue, and the length of time in which the circulating IL-6 is actually elevated, that is, a chronic response versus a somewhat brief increase that returns to baseline [33].

Skeletal muscle cells are at the same time a source and a target of IL-6 [10]. It has been shown that, with exercise and under physiological conditions, skeletal muscle-produced IL-6 can be elevated in the circulation for several hours and that the autocrine effects of IL-6 contribute to activation of the Stat3-SOCS3 signaling and to the positive regulation of the myogenic differentiation [10, 33]. By contrast, under pathologic conditions, including cancer cachexia, muscular dystrophy, and chronic inflammatory diseases, there is a chronic (i.e., long lasting) elevation of the circulating IL-6; although Stat3-SOCS3 signaling is also activated in the target skeletal muscle tissue under these pathologic conditions, the autocrine loop of regulation in the action of the cytokine is totally lost. It is possible that different intracellular signaling pathways are activated and this may explain why IL-6 can function both as positive and negative regulator of the myogenesis in physiological or in pathological conditions.

In skeletal muscle, AKT plays a very central role in the control of both muscle protein synthesis, via mTOR, and protein degradation, via the transcription factors of the FoxO family [19, 29]. However, in this study we found that rIL-6 did not directly affect AKT expression and activity during C2C12 myogenic differentiation (Figure 5).

Instead, we found that rIL-6 induced the concomitant downmodulation of two signaling axes which are downstream to AKT and are important for the regulation of the protein synthesis, namely, p90RSK/eEF2 and mTOR/p70S6K (see Figure 5 and [19, 21, 23, 29]).

In muscle cells, activated p90RSK phosphorylates and inactivates the elongation factor 2 kinase (eEF2K); inhibition of eEF2K activity results in a decrease in the inhibitory phosphorylation of eEF2 [21]. Indeed, only the dephosphorylated form of eEF2 is active and can catalyze the translocation of the peptidyl-tRNA from the A site to the P site on the ribosome and finally promote the protein synthesis [21] (see Figure 6(d)).

Activated p90RSK1 can also promote the protein synthesis acting on mTOR/p70S6K1 pathway independently of AKT activation [23] (Figure 6(d)). In fact, p90RSK1 has been found to interact with and phosphorylate the protein tuberin in a specific regulatory site and this phosphorylation inhibits the function of the tuberin-hamartin complex, resulting in increased mTOR signalling to p70S6K1 [9]. Finally, eEF2K activity can also be regulated by p70S6K; this latter kinase can in fact phosphorylate eEF2K and, as p90RSK1, can inhibit its activity [21].

It is evident that destabilization of this very intricate network of signaling pathways by the exogenous IL-6 (i.e., by the chronic elevation of circulating IL-6) can contribute to the decrease of the muscle protein synthesis and the promotion of muscle wasting (Figure 6(d)).

Our findings thus identify the p90RSK/eEF2 and mTOR/p70S6K axes as new potential targets of IL-6 activity on the myogenic program.

## 5. Conclusions

This study clarified the possible role of the IL-6 in the induction of skeletal muscle wasting. We show that rIL-6 impairs C2C12 myogenic differentiation, activates the Stat3-SOCS3 signaling pathway, and induces the downmodulation of p90RSK/eEF2 and mTOR/p70S6K axes, which are important to stimulate the protein synthesis and in the maintenance of the differentiated myogenic phenotype. Exogenous rIL-6 does not affect the expression levels and activation of AKT, a kinase essential to promote protein synthesis and cell survival and to block protein degradation in skeletal muscle cells.

Moreover, in this study we discuss how the pleiotropic cytokine IL-6 can contribute to the positive regulation of the myogenic differentiation under physiological conditions and to the negative regulation of the myogenic phenotype under some pathological circumstances.
